# Psychosocial health and quality of life in ICSI and naturally conceived adolescents: a cross-sectional comparison

**DOI:** 10.1007/s11136-023-03382-5

**Published:** 2023-03-16

**Authors:** N. Eisemann, M. Schnoor, E. Rakusa, C. C. Braren-von Stülpnagel, A. Katalinic, M. Ludwig, B. Sonntag, A. K. Ludwig, S. A. Elsner

**Affiliations:** 1 Institute of Social Medicine and Epidemiology, University of Luebeck, Ratzeburger Allee 160, 23562 Lübeck, Germany; 2 German Center for Neurodegenerative Diseases (DZNE), Bonn, Germany; 3 German Center for Health Services Research in Dermatology (CVderm), Institute for Health Services Research in Dermatology and Nursing (IVDP), University Medical Center Hamburg-Eppendorf, Hamburg, Germany; 4 SYNLAB Holding Deutschland GmbH, Augsburg, Germany; 5 amedes MVZ Hamburg GmbH, Facharztzentrum Für Kinderwunsch, Pränatale Medizin, Endokrinologie und Osteologie, Hamburg, Germany; 6 Praxis Für Frauengesundheit Und Pränatalmedizin, Hamburg, Germany

**Keywords:** Intracytoplasmic sperm injection, Adolescence, Quality of life, Behaviour, Psychosocial health, Disclosure

## Abstract

**Purpose:**

Psychosocial health (PH) and quality of life (QoL) are important health outcomes. We compared PH and QoL of adolescents conceived with intrazytoplasmatic sperm injection (ICSI) and of naturally conceived controls. The impact of disclosure of ICSI-conception on QoL and PH was quantified.

**Methods:**

The cross-sectional sample consisted of 545 ICSI-conceived adolescents and 427 unmatched singleton controls aged 14–18 years. Adolescents reported PH with the ‘Strengths and Difficulties Questionnaire’ (low values indicating high PH), and QoL with the KINDL questionnaire (high values indicating high QoL). Because of clustering of multiples within families, adjusted linear regressions with generalized estimating equations were used to compare ICSI- and naturally conceived adolescents. Missing values were treated by multiple imputation. Minimal importance was defined as half a standard deviation.

**Results:**

Both ICSI and control adolescents had high PH (low mean ‘total difficulties’ score: 9 of 40) and high QoL (mean ‘total KINDL’ score: 75 of 100). Differences were generally in favour of the ICSI group. Significant differences occurred for ‘impact of behavioural problems’ (*p* = 0.033), the ‘total KINDL’ score (*p* = 0.021) and the dimensions ‘physical wellbeing’ (*p* = 0.031) and ‘school’ (*p* = 0.005), but all differences were far below minimal importance. About 80% of ICSI adolescents were informed about their mode of conception. PH and QoL were slightly higher in informed adolescents; behavioural difficulties (‘total behavioural problems’ and ‘conduct problems’) were significantly lower (*p* = 0.013 and *p* = 0.003), behavioural strengths (‘prosocial behaviour’) and ‘physical QoL’ significantly higher (*p* = 0.004 and *p* = 0.018), but differences remained clearly below minimal importance.

**Conclusions:**

Our results are reassuring for parents using ICSI and their children. Speaking openly about an ICSI conception in the family may be beneficial.

**Supplementary Information:**

The online version contains supplementary material available at 10.1007/s11136-023-03382-5.

## Plain English summary

While there is an increasing body of evidence regarding potential physical differences between children conceived with intracytoplasmic sperm injection (ICSI) and naturally conceived children, less is known about differences in psychosocial health or quality of life. Although adolescence is a time of radical changes in psychological development, studies on the psychosocial health and quality of life of ICSI adolescents above an age of 14 years are currently completely lacking. In addition, it has not been studied so far if a disclosure of the ICSI conception is related to psychosocial health and quality of life in ICSI adolescents.

This manuscript reports findings from a large cross-sectional study in Germany which includes 545 ICSI conceived adolescents and 427 naturally conceived peers, all aged between 14 and 18 years. Overall, the averages of psychosocial health and quality of life were slightly better in ICSI adolescents than in the controls, but far below minimal importance. ICSI adolescents who were informed about their conception had slightly higher psychosocial health and quality of life than their uninformed counterparts, but again all differences remained far below minimal importance.

Our results are very reassuring for parents which are using ICSI for reproduction and for their offspring.

## Introduction

Assisted reproductive technologies (ARTs) include all interventions involving the in vitro treatment of both human egg and sperm or of embryos for the purpose of reproduction [1]. Apart from a small minority, these treatments are in vitro fertilisations (IVFs) with embryo transfer. Today, about 70% of IVFs are performed with intracytoplasmatic sperm injection (ICSI) [2, 3].

More than 330,000 childbirths resulted from ART treatments in 2017 in the countries of the ART World Registry [3], and another 310,000 in China [4]. In Germany, the annual number of births after ICSI have stabilized over the last five years (approx. 12,500 births per year, corresponding to 1.9% of all born children) [2].

Similar to the conventional IVF, the introduction of ICSI raised questions about the safety for the children conceived using this technology. The possible risk from the exposure of the fertilized egg to an artificial nutrient solution during the periconceptional time—the most important window during which all cells are fully exposed to environmental conditions [5]—and the issue of multiple pregnancies and pre-term births is common for conventional IVF and IVF with ICSI. In the case of ICSI, abnormal sperm of a subfertile father, the by-passing of the natural process of sperm selection by choosing a single sperm for fertilization, and the penetration of the egg by a micro-pipette may further influence the child’s health. In addition to these biological factors, the group of ICSI parents has certain characteristics that may affect the children’s health outcomes, especially the psychosocial ones: Parents who received fertility treatment tend to be older, better educated, and have a higher income compared to parents with naturally conceived (NC) children [6], which might lower the risk of developing psychosocial problems [7]. On the other hand, they share the experience of infertility and the efforts to overcome it. Overprotectiveness and excessive expectations of parenthood and the child’s achievements can have a negative impact on the child's psychosocial development [8, 9].

There is well-supported evidence on perinatal health of ICSI-conceived children and a wide range of studies on health in childhood, but studies on health in adolescence are still scarce [10]. So far, they refer primarily to physical health [11–16]. Psychosocial health, which includes mental, emotional, social, and spiritual dimensions, is no less important for a fulfilled life with social participation and a good quality of life. With a growing number of ICSI children reaching adolescence, it is increasingly important to fill this research gap.

Overall, evidence regarding the psychosocial health (PH) and quality of life (QoL) of ICSI adolescents and the effect of disclosure of an ICSI-conception on these outcomes is lacking. This study will compare data from a large sample of ICSI adolescents to NC control adolescents to generate insight into these questions.

## Methods

### The ICSI study

**Fig. 1:**
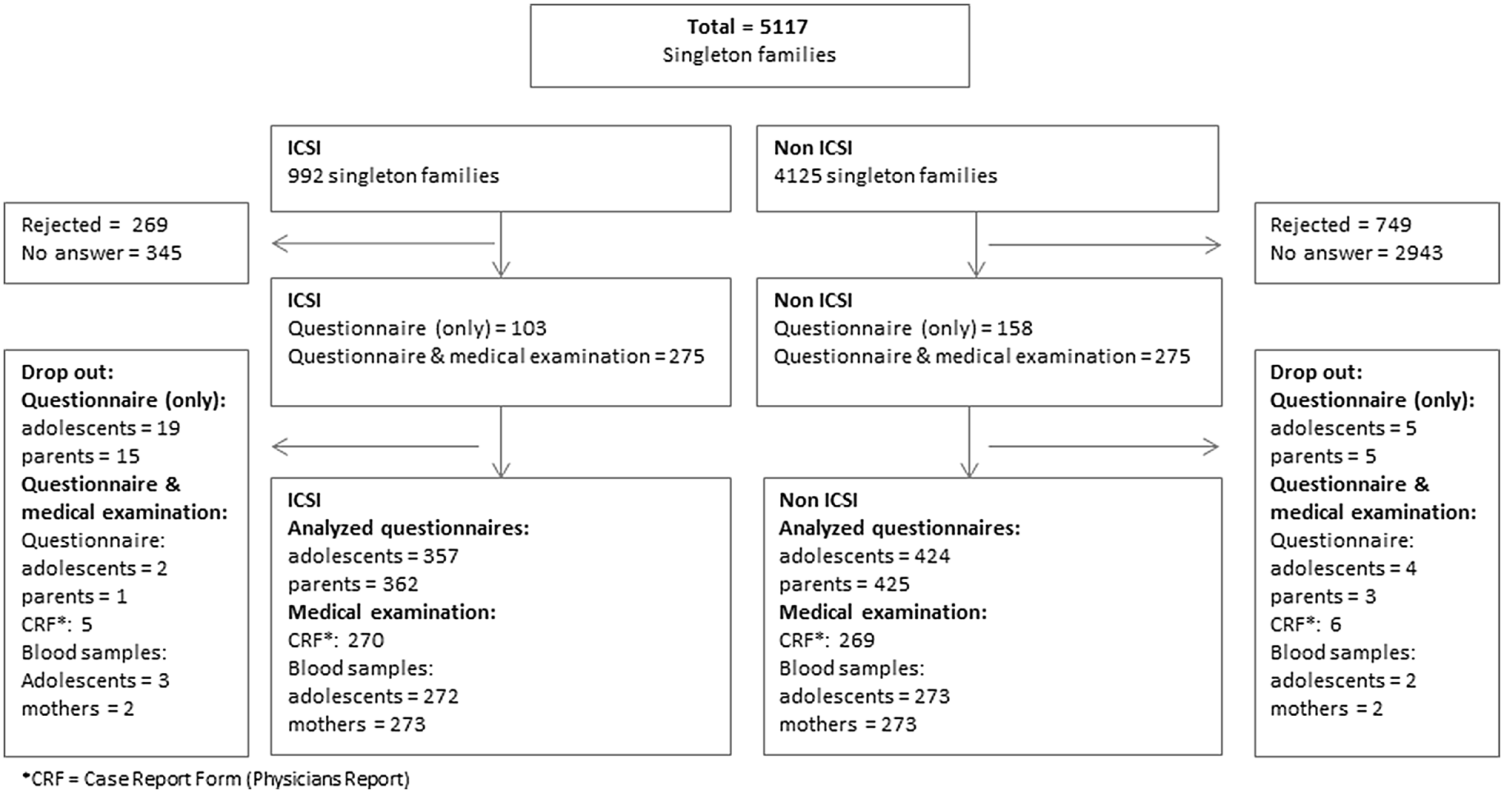
Flow chart of the study sample

The ICSI study is a prospective controlled study performed throughout Germany. The initial ICSI cohort was recruited in early pregnancy before 16 weeks of gestation [11]. After a follow-up examination after birth and at the age of 4–6 [17], a third follow-up took place at the age of 14–18 (Fig. 1). As controls, a new random sample of singletons was recruited via German registration offices. More information can be found in [15, 16]. This analysis is based on questionnaire data about QoL and PH from follow-up III.

This study was performed in line with the principles of the Declaration of Helsinki. The study protocol was approved by the ethical review board of the University of Luebeck, Germany (Reference Number: 13-193). Parents and adolescents gave written informed consent.

### Outcomes and other variables

The main outcomes of this analysis were self-reported PH, measured by the German version of the Strengths and Difficulties Questionnaire (SDQ) [18] and self-reported QoL, measured by the KINDL questionnaire [19, 20].

The SDQ is a brief behavioural screening questionnaire for 3- to 16-year-olds, which is commonly used to assess PH in similar studies [21–23]. The 25 items are aggregated into five dimensions, four of which measure behavioural difficulties: ‘conduct problems’, ‘hyperactivity’, ‘emotional problems’ and ‘peer problems’. Values range between 0 and 10. Summed up they result in the ‘total difficulty score’. The fifths dimension, the ‘pro-social behaviour’, is about behavioural strengths and not part of the total score. In addition, the perceived ‘impact’ of any behavioural difficulties is measured, which ranges between 0 and 10. A higher score indicates a greater degree of behavioural difficulties, pro-social behaviour or higher impact of difficulties on life, respectively.

The KINDL questionnaire is a short, methodically tested and flexible instrument for health-related QoL in 3- to 17-year old. Its 24 items are aggregated into six dimensions (‘physical well-being’, ‘emotional well-being’, ‘self-esteem’, ‘family’, ‘friends’ and ‘school well-being’) and a ‘total score’. All scores, including the total score, range between 0 and 100. The higher the value, the higher the QoL.

The SDQ and the KINDL questionnaires were answered by the adolescents themselves (self-report) and by their parents (proxy-report). We focused on the adolescents' self-reports, and only used the parents' proxy-reports—together with other auxiliary variables—to impute missing data (see statistical methods).

Although the SDQ and the KINDL were developed for adolescents up to 16 and 17 years, respectively, they were administered to all adolescents up to 18 years to keep the reports of all adolescents on comparable scales.

Adolescents also reported data on sociodemographics (age, sex, having a twin or triplet, secondary school type), lifestyle behaviour (physical exercise, smoking, alcohol consumption) and health (body mass index (BMI), existence of any severe physical or mental diseases). BMI was age- and sex-standardized to a Z-score (German reference population: mean 0, standard deviation 1) using the KIGGS reference data [24] and categorized as underweight (< 5th percentile), normal (5th to < 85th percentile), overweight (≥ 85th percentile) and obese (≥ 95th percentile).

Mothers were asked about their socioeconomic status (SES) and parental factors (age at birth, educational status, living single or with a partner, net household income) and, if applicable, about disclosure of the mode of conception to the ICSI adolescent.

### Minimal important difference (MID)

Results from group comparisons should always consider both the significance and the importance of estimated differences. Ideally, our definition of a minimal important difference (MID) in QoL or behavioural difficulties would be based on the adolescents' perspective [25]. As there is no such information available, we used the definition of half a standard deviation, as this was found to be often close to the minimal important difference reported by patients or other persons who were studied [26].

### Statistical methods

Characteristics of the two study groups and for the ICSI subgroups ‘singletons’, ‘twins’ and ‘triplets’ were analyzed descriptively, and the ICSI and NC groups compared using Welch and Chi-squared tests. The distributions of the outcomes in the ICSI and the NC group were presented in box-and-whisker plots.

Linear regression models for PH and QoL were fitted to compare the two groups. First, the overall effect of ICSI was assessed from unadjusted models (model 1). In order to isolate the direct ICSI effect from confounding effects due to parental and socioeconomic factors, the models were then adjusted for the age of the mother at birth, the highest school degree of the mother, if the mother is living single or with a partner and the family’s monthly net income (model 2). Following a recent recommendation for control of covariates [27], adolescent factors that may affect PH and QoL but occur far after the conception (all remaining variables, see Table 1) were added to the regression (model 3). In order to account for the clustering of twins or triplets within a family, generalised estimating equations (GEEs) were used. We conducted sensitivity analyses where only singletons of the ICSI group were included.

**Table 1:** Characteristics of the study population

	Controls	ICSI-conceived adolescents				p-value	
	Singletons^a^ (*N* = 427)	Total (*N* = 545)	Singetons (*N* = 366)	Twins (*N* = 161)	Triplets (*N* = 18)	Total	Only singletons
Age. *N* (%)						< 0.001^**b**^	0.739^**b**^
14 years	25 (5.9)	–	–	–	–		
15 years	112 (26.5)	73 (14.0)	72 (20.3)	1 (0.7)	–		
16 years	160 (37.8)	315 (60.6)	231 (65.3)	78 (52.7)	6 (33.3)		
17 years	119 (28.1)	127 (24.4)	50 (14.1)	65 (43.9)	12 (66.7)		
18 years	7 (1.7)	5 (1.0)	1 (0.3)	4 (2.7)	–		
Missing of total	*4 (0.9)*	*25 (4.6)*	*12 (4.6)*	*13 (8.1)*	–		
Sex. *N* (%)						0.977^**c**^	0.978^**c**^
Female	222 (52.0)	285 (52.3)	189 (51.6)	86 (53.4)	10 (55.6)		
Male	205 (48.0)	260 (47.7)	177 (48.4)	75 (46.6)	3 (44.4)		
Secondary school. *N* (%)						0.168^**d**^	0.011^**d**^
Highest academic level (12th or 13th grade)	280 (71.4)	339 (72.8)	238 (72.8)	93 (75.6)	8 (50.0)		
Intermediate academic level (10th grade)	41 (10.5)	63 (13.5)	54 (16.5)	8 (6.5)	1 (6.2)		
Lowest academic level (9th grade)	7 (1.8)	8 (1.7)	4 (1.2)	1 (0.8)	3 (18.8)		
Comprehensive school	47 (12.0)	39 (8.4)	22 (6.7)	15 (12.2)	2 (12.5)		
School for children with special needs	1 (0.3)	5 (1.1)	3 (0.9)	2 (1.6)	–		
Other school	16 (4.1)	12 (2.6)	6 (1.8)	4 (3.3)	2 (12.5)		
Missing of total. N (%)	*35 (8.2)*	*79 (14.5)*	*39 (10.7)*	*38 (23.6)*	*2 (11.1)*		
Physical exercise. *N* (%)						0.199^**c**^	0.380^**c**^
None	43 (10.3)	36 (7.1)	29 (8.3)	6 (4.3)	1 (5.6)		
Once per week	68 (16.2)	83 (16.3)	58 (16.5)	21 (15.0)	4 (22.2)		
2–3 times a week	187 (44.6)	255 (50.1)	176 (50.1)	69 (49.3)	10 (55.6)		
More often	121 (28.9)	135 (26.5)	88 (25.1)	44 (31.4)	3 (16.7)		
Missing of total. N (%)	*8 (1.9)*	*36 (6.6)*	*15 (4.1)*	*21 (13.0)*	–		
Smoking. *N* (%)						0.246^**c**^	0.288^**c**^
Yes	44 (10.5)	41 (8.1)	28 (8.0)	10 (7.1)	3 (16.7)		
No	376 (89.5)	468 (91.9)	323 (92.0)	130 (92.9)	15 (83.3)		
Missing of total. N (%)	*7 (1.6)*	*36 (6.6)*	*15 (4.1)*	*21 (13.0)*	–		
Alcohol consumption. *N* (%)						< 0.001^**c**^	0.153^**c**^
Yes	247 (58.8)	354 (69.6)	225 (64.1)	113 (80.7)	16 (88.9)		
No	173 (42.2)	155 (30.5)	126 (35.9)	27 (19.3)	2 (11.1)		
Missing of total. N (%)	*7 (1.6)*	*36 (6.6)*	*15 (4.1)*	*21 (13.0)*	–		
Body mass index. Mean (SD)							
Original value	21.2 (3.3)	21.2 (3.4)	21.3 (3.4)	20.6 (2.4)	24.1 (7.0)	0.781^**b**^	0.856^**b**^
Z-score	− 0.65 (1.1)	− 0.72 (1.1)	− 0.66 (1.1)	− 0.93 (1.0)	− 0.25 (1.1)		
Categories. *N* (%)							
Underweight	53 (12.8)	70 (13.7)	45 (12.9)	23 (15.8)	2 (11.8)		
Normal weight	344 (83.1)	411 (80.6)	280 (80.2)	122 (83.6)	11 (64.7)		
Overweight	13 (3.1)	20 (3.9)	19 (5.4)	1 (0.7)	–		
Obesity	4 (1.0)	9 (1.8)	5 (1.4)	–	4 (23.5)		
Missing of total. N (%)	*13 (3.0)*	*35 (6.4)*	*17 (4.6)*	*15 (9.3)*	*1 (5.6)*		
Severe physical disease. *N* (%)						0.555^**c**^	0.618^**c**^
Yes, one or more	48 (11.3)	68 (12.8)	46 (12.7)	18 (11.8)	4 (22.2)		
None	377 (88.7)	465 (87.2)	316 (87.3)	135 (88.2)	14 (77.8)		
Missing of total. N (%)	*2 (0.5)*	*12 (2.2)*	*4 (1.1)*	*8 (5.0)*	–		
Severe psychological disease. *N* (%)						0.999^**c**^	0.999^**c**^
Yes, one or more	38 (8.9)	48 (9.0)	33 (9.1)	13 (8.5)	2 (11.1)		
None	387 (91.1)	485 (91.0)	329 (90.9)	140 (91.5)	16 (88.9)		
Missing of total. N (%)	*2 (0.5)*	*12 (2.2)*	*4 (1.1)*	*8 (5.0)*	–		
Age of mother at birth. Mean (SD)	31.6 (4.6)	33.7 (3.7)	33.7 (4.0)	33.6 (3.4)	34.0 (2.3)	< 0.001^**b**^	< 0.001^**b**^
Missing of total. N (%)	*1 (0.2)*	*1 (0.2)*	*1 (0.3)*	*-*	*-*		
Highest school degree of mother. *N* (%)						< 0.001^**e**^	< 0.001 ^**e**^
Highest academic level (12th or 13th grade)	285 (67.1)	238 (44.7)	53 (41.7)	78 (51.0)	9 (50.0)		
Intermediate academic level (10th grade)	118 (27.7)	240 (45.0)	264 (45.3)	67 (43.8)	9 (50.0)		
Lowest academic level (9th grade)	20 (4.7)	48 (9.0)	40 (11.0)	8 (5.2)	–		
Other school	2 (0.5)	6 (1.1)	6 (1.7)	–	–		
None	–	1 (0.2)	1 (0.3)	–	–		
Missing of total. N (%)	*2 (0.2)*	*12 (2.2)*	*4 (2.5)*	*8 (5.0)*	–		
Family status of mother. *N* (%)						< 0.001^**c**^	0.002 ^**c**^
Living as single parent	87 (20.5)	62 (11.4)	44 (12.2)	18 (11.8)	0 (0.0)		
Living with a partner	338 (79.5)	471 (88.6)	318 (87.8)	135 (88.2)	18 (100.0)		
Missing of total. N (%)	*2 (0.5)*	*12 (2.2)*	*4 (1.1)*	*8 (5.0)*	*-*		
Monthly net household income. *N* (%)						< 0.001^**c**^	0.018 ^**c**^
Less than 1500 Euro	20 (4.8)	4 (0.8)	4 (1.2)	–	–		
1500–3000 Euro	127 (30.5)	153 (30.4)	106 (31.3)	38 (25.5)	9 (60.0)		
More	269 (64.7)	346 (68.8)	229 (67.6)	11 (74.5)	6 (40.0)		
Missing of total. N (%)	*11 (2.6)*	*42 (7.7)*	*27 (7.4)*	*12 (7.4)*	*3 (20.0)*		

^a^Only singletons were included as controls

^b^Welch Two Sample t-test

^c^Chi-squared test

^d^Chi-squared test after combining School for children with special needs with Lowest academic level (because of small expected cell frequencies)

^e^Chi-squared test after combining None with Lowest academic level and excluding Other school (because of small expected cell frequencies)

Although only 5.1% of values were missing overall (1148/22,356), there were approximately one third of adolescents (34.7% of 972) who had at least one missing value. Exclusion of these adolescents would have resulted in a complete-case analysis with 635 study participants (65.3%). In order to reduce potential selection bias due to missing data, we retained all study participants in the analysis with all data that was reported, and applied multiple imputation under fully conditional specification [28]. We assumed that missingness occurred at random. As 1. questionnaire items of the same domain are correlated, 2. proxy and self-reports are correlated and 3. the relationship between outcomes and ICSI/control group is to be analysed, all the respective variables were selected to serve as a set of auxiliary variables for predicting the imputations of each variable with missing values. If present, variables measuring something very similar were also selected (for example, psychological well-being and presence of psychological disease). For deterministic relations such as the relation between total score and individual items, passive imputation was applied. Ten imputed data sets were created and results pooled using Rubin’s rule. Due to late convergence of imputation chains for some variables, 50 iterations were allowed. Using logistic regression for imputation of binary variables and predictive mean matching for all others, all imputations had plausible values.

We further analyzed whether the disclosure of ICSI-conception had an influence on the QoL or PH in the ICSI adolescents. For this, the subset of multiply imputed data of the ICSI group was used. GEEs without and with adjustment for socioeconomic, parental and adolescent factors were fitted.

All analyses are of explorative nature. They were conducted with R 4.1.3 [29].

## Results

In total, 545 ICSI-conceived adolescents from 453 families and 427 NC controls were included. Some of the group characteristics differed significantly (Table 1): ICSI adolescents were on average 2.4 months older than controls (16.6 vs. 16.2 years), due to older age in twins (17.0 years) and triplets (17.1 years). Alcohol consumption was more prevalent in ICSI than in control adolescents, with proportions being higher for twins and triplets. ICSI mothers were on average two years older at birth, slightly less educated, and less often living single. The monthly net household income was generally higher in the ICSI group.

### Psychosocial health

The observed ‘total difficulties’ score of the SDQ was on average slightly lower in ICSI adolescents than in controls (8.6, SD 4.5 vs. 9.2, SD 4.4), meaning there was a lower extent of behavioural difficulties and thus a higher PH in the ICSI group (Table 2). However, the difference did not reach the MID (half SD = 2.2). The same pattern occurred for ‘emotional problems’ and ‘hyperactivity’. As for the QoL, a gradient was found in all four difficulty dimensions, with behavioural difficulties increasing in twins and finally triplets.

**Table 2:** Description of psychosocial health and quality of life in ICSI-conceived adolescents and naturally conceived controls (original observations)

	NC control			ICSI							
				Total		Singletons		Twins		Triplets	
	*N*	Mean (SD)	MID	*N*	Mean (SD)	*N*	Mean (SD)	*N*	Mean (SD)	*N*	Mean (SD)
Psychosocial health: SDQ											
Total difficulties (0–40 scale)	415	9.2 (4.4)	± 2.2	505	8.6 (4.5)	349	8.3 (4.5)	138	9.1 (4.4)	18	10.0 (5.3)
Emotional problems (0–10 scale)	415	3.0 (2.4)	± 1.1	505	2.7 (2.2)	349	2.7 (2.1)	138	2.9 (2.2)	18	3.1 (3.0)
Conduct problems (0–10 scale)	415	1.3 (1.1)	± 0.5	505	1.3 (1.2)	349	1.2 (1.1)	138	1.5 (1.4)	18	1.6 (1.1)
Peer problems (0–10 scale)	415	2.0 (1.6)	± 0.8	505	2.0 (1.6)	349	2.0 (1.6)	138	2.0 (1.8)	18	2.1 (1.7)
Hyperactivity (0–10 scale)	415	2.9 (1.9)	± 1.0	505	2.6 (2.0)	349	2.5 (2.0)	138	2.7 (2.0)	18	3.2 (1.9)
Prosocial behaviour (0–10 scale)	415	8.1 (1.7)	± 0.9	505	8.1 (1.8)	349	8.1 (1.8)	138	8.1 (1.7)	18	7.3 (2.2)
Impact (0–10 scale)	420	0.4 (1.0)	± 0.5	509	0.2 (0.8)	352	0.2 (0.7)	139	0.3 (0.9)	18	0.3 (0.7)
Quality of life: KINDL (0–100 scale)											
Total score	418	74.3 (11.3)	± 5.7	521	75.7 (11.9)	355	76.5 (11.6)	148	74.7 (11.7)	18	68.3 (16.4)
Physical wellbeing	375	71.6 (19.8)	± 35.8	462	74.3 (18.8)	315	74.9 (19.1)	132	73.6 (17.5)	15	68.2 (22.1)
Psychological wellbeing	396	83.1 (12.7)	± 6.3	485	84.1 (13.0)	331	85.5 (12.0)	137	81.6 (14.5)	17	77.7 (15.4)
Self esteem	422	63.0 (17.8)	± 31.5	523	64.5 (17.4)	357	65.9 (17.2)	148	63.0 (17.0)	18	50.3 (16.5)
Family	413	86.7 (15.2)	± 7.6	512	86.7 (16.1)	349	87.2 (16.2)	145	86.6 (15.6)	18	76.9 (18.2)
Friends	422	75.8 (15.2)	± 7.6	520	76.9 (16.1)	356	76.8 (16.2)	146	77.6 (15.6)	18	72.8 (18.2)
School	378	67.3 (19.0)	± 9.5	478	71.0 (17.2)	325	72.2 (17.2)	136	68.4 (17.7)	17	66.8 (20.9)

ICSI: Adolescents conceived with intrazytoplasmatic sperm injection

NC: Adolescents conceived naturally

SD: Standard deviation

MID: Minimal important difference, defined as 0.5 times the SD

Both groups showed high behavioural strengths (mean ‘pro-social behaviour’ of 8.1 in both groups) and a low impact of behavioural difficulties on the adolescents’ lives (‘impact’ of << 1 in both groups).

Group differences remained very similar after multiple imputation of missing values and accounting for the clustering of multiples, and they remained similar after adjustment for socioeconomic and parental factors and further adjustment for adolescent factors (Table 3). Significantly better scores for the ICSI group were found for ‘impact of behavioural problems’, but the difference of − 0.1 was far below the MID of ± 0.5.

**Table 3:** Comparison of psychosocial health and quality of life in ICSI-conceived adolescents versus naturally conceived controls using adjusted generalized estimating equations after multiple imputation—singletons and multiples

	Model 1: ICSI − NC No adjustment			Model 2: ICSI − NC Adjustment for socioeconomic and parental factors^a^			Model 3: ICSI − NC Adjustment for socioeconomic and parental factors^a^ and adolescent factors^b^		
	Mean	95%-CI	*p*	Mean	95%-CI	*p*	Mean	95%-CI	*p*
Psychosocial health: SDQ									
Total difficulties (0–40 scale)	− 0.5	− 1.1 to 0.1	0.138	− 0.4	− 1.0 to 0.3	0.275	− 0.4	− 1.0 to 0.3	0.281
Emotional problems (0–10 scale)	− 0.3	− 0.6 to 0.04	0.087	− 0.3	− 0.6 to 0.1	0.151	− 0.3	− 0.6 to 0.04	0.085
Conduct problems (0–10 scale)	0.0	− 0.1 to 0.2	0.925	0.0	− 0.1 to 0.2	0.702	0.1	− 0.1 to 0.2	0.546
Peer problems (0–10 scale)	0.1	− 0.1 to 0.4	0.208	0.1	− 0.1 to 0.4	0.305	0.1	− 0.1 to 0.4	0.278
Hyperactivity (0–10 scale)	**− 0.3**	**− 0.5 to − 0.01**	**0.040**	− 0.2	− 0.5 to 0.1	0.158	− 0.2	− 0.4 to 0.1	0.178
Prosocial behaviour (0–10 scale)	0.0	− 0.3 to 0.2	0.773	0.0	− 0.3 to 0.2	0.831	− 0.1	− 0.3 to 0.2	0.567
Impact (0–10 scale)	**− 0.2**	**− 0.3 to − 0.05**	**0.005**	**− 0.1**	**− 0.2 to − 0.01**	**0.028**	**− 0.1**	**− 0.2 to − 0.01**	**0.033**
Quality of life: KINDL (0–100 scale)									
Total score	**1.5**	**0.1 to 3.0**	**0.040**	1.6	− 0.03 to 3.2	0.055	**1.8**	**0.3 to 3.4**	**0.021**
Physical wellbeing	**2.7**	**0.2 to 5.2**	**0.037**	2.7	− 0.1 to 5.4	0.062	**2.9**	**0.3 to 5.6**	**0.031**
Psychological wellbeing	1.0	− 0.8 to 2.8	0.281	0.8	− 1.1 to 2.7	0.427	1.0	− 0.8 to 2.8	0.288
;Self esteem	1.7	− 0.6 to 4.0	0.142	2.1	− 0.3 to 4.6	0.092	2.3	− 0.1 to 4.7	0.057
Family	− 0.2	− 2.2 to 1.7	0.806	− 0.3	− 2.4 to 1.8	0.801	− 0.3	− 2.3 to 1.8	0.791
Friends	1.1	− 1.0 to 3.1	0.303	1.4	− 0.8 to 3.6	0.209	1.5	− 0.7 to 3.7	0.174
School	**3.3**	**0.8 to 5.7**	**0.009**	**3.3**	**0.7 to 6.0**	**0.015**	**3.7**	**1.1 to 6.4**	**0.005**

ICSI − NC: difference between adolescents conceived with intrazytoplasmatic sperm injection and adolescents conceived naturally

^a^Parental and socioeconomic factors were age of the mother at birth, the highest school degree of mother, if the mother is living single or with a partner and the family’s monthly net income

^b^Adolescent factors were age at time of study, sex, type of secondary school, physical exercise, smoking, alcohol consumption, body mass index category, presence of severe physical disease and presence of severe psychological disease

**Bold** indicates that the 95% confidence interval does not contain 0

### Quality of life

Differences in the QoL between ICSI and control adolescents were small but partly significant (Tables 2, 3).

A mean ‘total KINDL’ score of 74.3 (SD 11.3) was observed in the control group. The whole ICSI group scored negligibly higher (mean 75.7, SD 11.9). When stratifying by the number of siblings, singletons had generally higher scores than twins and triplets (Table 2). After imputation of missing values, accounting for clustering and full adjustment for covariates, ICSI adolescents had on average a 1.8 point higher ‘total KINDL’ score than controls (95%-CI 0.3 to 3.4) (Table 3).

Similar results were found in all dimensions. Average descriptive values varied between 65.9 (‘self-esteem’) and 85.5 (‘psychological well-being’) in ICSI singletons and between 64.5 (‘self-esteem’) and 81.1 (‘psychological well-being’) in controls. Again, a gradient was found in all dimensions, with QoL decreasing with the number of siblings. The unadjusted model as well as the fully adjusted model identified significantly higher QoL regarding ‘total score’, ‘physical wellbeing’ and ‘school’ in ICSI than in control adolescents, but all differences remained clearly below the respective MID thresholds shown in Table 2.

### Sensitivity analyses

When excluding all multiples of the ICSI group, the advantages of the ICSI group increased slightly, especially in the unadjusted model (Online Resource: “Supplemental table.pdf”). Although the differences were now more often significant, they all remained far below the limit of minimal importance. A complete-case analysis gave results similar to the main analysis, with slightly weaker effects and larger p-values for QoL in ICSI vs. control adolescents.

### Disclosure of ICSI-conception

About 80% of the ICSI adolescents were informed about their mode of conception.

The PH was slightly better in informed adolescents, both without and with adjustment for covariates; the consistently lower mean scores of the ‘total difficulties’ score and its four subscales were all below the MID, but significant for ‘total difficulties’, ‘conduct problems’ and ‘prosocial behaviour’. No difference was found for the ‘SDQ impact’.

QoL was slightly higher in informed adolescents, but reached neither the MID nor significance (mean differences of dimensions ranged between 0.5 and 4.3 points, Table 4). After adjustment for covariates, the ‘physical wellbeing’ dimension became significant.

**Table 4:** Comparison of psychosocial health and quality of life in ICSI adolescents who are versus are not informed about being conceived with ICSI, using adjusted generalized estimating equations after multiple imputation

	Difference of informed vs. uninformed ICSI adolescents					
	Model 1: no adjustment			Model 2: adjustment for socioeconomic, parental and adolescent factors^a^		
	Mean	95%-CI	*p*	Mean	95%-CI	*p*
Psychosocial health: SDQ						
Total difficulties (0–40 scale)	**− 1.4**	**− 2.6 to − 0.2**	**0.019**	**− 1.3**	**− 2.5 to − 0.3**	**0.013**
Emotional problems (0–10 scale)	− 0.1	− 0.7 to 0.5	0.748	0.0	− 0.6 to 0.5	0.883
Conduct problems (0–10 scale)	**− 0.4**	**− 0.7 to − 0.2**	**0.002**	**− 0.4**	**− 0.7 to − 0.1**	**0.003**
Peer problems (0–10 scale)	− 0.4	− 0.9 to 0.1	0.105	− 0.4	− 0.9 to 0.02	0.059
Hyperactivity (0–10 scale)	**− 0.5**	**− 1.0 to − 0.004**	**0.048**	**− 0.6**	**− 1.1 to − 0.05**	**0.032**
Prosocial behaviour (0–10 scale)	**0.7**	**0.2 to 1.2**	**0.009**	**0.7**	**0.2 to 1.2**	**0.004**
Impact (0–10 scale)	0.0	− 0.1 to 0.2	0.653	0.1	− 0.1 to 0.2	0.477
Quality of life: KINDL (0–100 scale)						
Total score	2.5	− 0.3 to 5.2	0.083	2.6	− 0.2 to 5.4	0.065
Physical wellbeing	4.2	− 0.1 to 8.6	0.058	**5.5**	**1.0 to 10.1**	**0.018**
Psychological wellbeing	3.0	− 0.2 to 6.2	0.068	2.6	− 0.7 to 5.9	0.124
;Self esteem	2.1	− 2.2 to 6.4	0.334	1.6	− 2.9 to 6.0	0.488
Family	3.0	− 1.2 to 7.2	0.154	2.8	− 1.0 to 6.5	0.145
Friends	1.0	− 3.0 to 5.1	0.613	0.9	− 3.2 to 5.1	0.657
School	0.5	− 4.3 to 5.3	0.834	0.6	− 4.2 to 5.4	0.805

^a^Socioeconomic, parental and adolescent factors were age of the mother at birth, the highest school degree of mother, if the mother is living single or with a partner, the family’s monthly net income, the adolescent’s age, sex, type of secondary school, physical exercise, smoking, alcohol consumption, body mass index category, presence of severe physical disease and presence of severe psychological disease

**Bold** indicates that the 95% confidence interval does not contain 0

## Discussion

This study provides for the first time data on the psychosocial health and quality of life of a large cohort of ICSI-conceived adolescents. Their average PH and QoL was good (mean ‘total difficulties’ score: 9 of 40, mean ‘total KINDL’ score: 75 of 100). When comparing ICSI to NC adolescents, the differences in the mean total scores and subscores were generally small and far below the MID, but indicating a slight tendency to better PH and QoL in the ICSI group. Some differences were statistically significant: ICSI adolescents had a significantly lower perceived ‘impact’ of behavioural difficulties, and a significantly higher QoL in the ‘total KINDL’ score, the ‘physical wellbeing’ and the ‘school’ dimension.

Barbuscia et al. (2019) cautioned that parental factors may impact and even reverse differences in PH between ART and NC children. Children and adolescents with a lower socioeconomic status are more frequently affected by mental disorders and low PH in terms of SDQ [7, 30]. Similarly, a higher social status can lead to higher overall QoL scores [31, 32]. Thus, we were careful to fit also regressions that adjusted for important parental factors and additionally regressions that adjusted for all covariates selected as potential confounders [27]. However, the estimated group means of PH and QoL barely shifted. Possibly parental and socioeconomic factors impact PH in adolescents less than in children. One other reason may lie in the smaller differences in the parental and socioeconomic factors of this study. Compared to the study by Barbuscia et al., ICSI mothers were also on average older at birth, less often living single and had more often a higher monthly net household income, but the differences in age and single mother proportion were smaller (2 vs. 4 years older, 9 vs. 12% less single mothers). The participating ICSI mothers were even on average slightly less educated than the participating NC mothers.

Although the adjustment for parental factors introduced only very small changes in the differences between ICSI and NC adolescents, the difference in the ‘total KINDL’ score and the ‘physical wellbeing’ dimension turned insignificant. However, further adjustment for adolescent factors undid these changes.

The remaining estimated advantages of the ICSI group may be due to chance, residual confounding or to actually superior outcomes—though of negligible size. The last explanation would fit with the results of a recent study in which—after adjustment—better QoL was found in young ART adults (22–35 years) than in their naturally conceived controls [33]. A study on PH in younger children (5–8 years old) found a higher risk for autism in ICSI children [34], but a recent study with children up to the age of 14 years indicated that any differences in PH vanish by the age of 14, and that the same patterns were found for all assisted reproduction methods, including ICSI [23].

As adolescents strive to develop their own personal identity and to detach themselves from their parents to gain more autonomy [35, 36], this developmental phase has a crucial impact on PH and QoL [37, 38]. Thus, findings in childhood must not necessarily translate into similar findings in adolescence. However, similar to our study, the QoL of young ICSI and NC children (5–8 years) was found to be similar, with a very slight tendency to better QoL in ICSI children [34]. A review about adolescents (11–18 years), that did not focus specifically on ICSI, also found no greater difficulties in psychological adjustment for IVF adolescents than for NC adolescents [39]. So far, it can be concluded that PH and QoL in ICSI adolescents are not inferior to that in NC adolescents.

Apart from the ICSI-conception itself, the knowledge about it may also influence PH and QoL. A disclosure may cause happiness because of feeling deeply wanted; or adolescents may resent the fact of being conceived ‘unnaturally’ and show more psychosocial problems and lower QoL. Evidence on this topic is currently lacking. A review found studies about consequences of conception disclosure only for families where the child was conceived with the help of egg donation or donor insemination [39]. The review reports that parent–adolescent relationships were of good quality in all families, but children who were informed about their mode of conception at young age reacted less negative than later in life. A side effect of early disclosure is that unintentional disclosure, which may cause negative reactions, is naturally prevented. It is suggested that the positive parent–adolescent relationships seen in families that have disclosed their use of reproductive donation may not result from the disclosure itself, but from a more open communication style [39]. As an ICSI disclosure (usually) does not change the perceived biological parent–child connection, even smaller effects on relationship, PH and QoL can be expected.

In fact, no difference in PH was found in the second ICSI follow-up study between children aged 5 to 6 years who were or were not informed about their conception mode [40]. At that time, only 2.3% of parents reported a disclosure. The others considered disclosure unimportant or wanted to protect the child from worry [40]. On the other hand, Colpin et al. found in 8- to 9-year-old IVF children that the informed ones had significantly more behavioural problems, though still in the normal range [22].

In the third ICSI follow-up study here, with adolescents aged 14 to 18 years, 80% of mothers reported a disclosure. Informed adolescents showed slightly higher PH and QoL than uninformed adolescents, but the differences remained far below the thresholds of minimal importance. Nevertheless, differences were significant for the ‘total difficulties’ score, the dimensions ‘conduct’ and ‘hyperactivity’ (only before adjustment for parental factors), the ‘pro-social behaviour’ and the QoL dimension ‘physical wellbeing’ (only after adjustment). All of these differences should be interpreted with caution due to their small size, but especially the difference in physical wellbeing. It is the dimension least related to psychological processing, and an impact of ICSI disclosure on this dimension seems least plausible. As one can expect that between 0 and 1 of the fourteen 95%-confidence intervals of this sub-analysis do not to contain the true difference, this finding appears to be a chance finding.

Assuming that the associations between PH and knowing about the ICSI-conception are small but real, the observational nature of the study still prohibits drawing any conclusions about the direction of causality. As mentioned by Ilioi and Golombok [39], a more open communication style may be the actual cause of both the disclosure and the higher PH.

This study comes with some limitations.

A selection bias towards families with a more favourable parental and socioeconomic background is suspected because of an unexpectedly high education of NC mothers and because of a comparison to the German norm population [41], which shows consistently superior PH and QoL outcomes for both study groups (difficulty scores of the SDQ were between -1 and 0 points lower and the ‘prosocial behaviour’ score 0.8 points higher, KINDL scores were between 0.6 and 6.8 points higher). Such a selection bias is not uncommon for observational studies. Notably, it does not hamper the adjusted comparison of the ICSI and NC adolescents.

Missing values, which can decrease the power of a study and introduce bias, are treated by multiple imputation. Further, although the inclusion of multiples in the ICSI group is an improvement over studies which focus solely on singletons, no multiples were recruited in the control sample. We found that multiples differ from singletons with regard to baseline characteristics and outcomes (PH and QoL were generally lower). Consequently, the exclusion of them from the NC group introduces bias, even though the bias is attenuated by the fact that multiples are born less frequently after natural conception than after ICSI. However, we can make a statement on the extent of the bias: The actual differences must lie between the results from the main analysis, which overestimate negative ICSI effects, and the supplementary results from the sensitivity analyses, which exclude also the ICSI multiples and overestimate the advantage of the ICSI group.

Overall, there are no indications that adolescents conceived with ICSI are at higher risk of an impaired PH or a lower QoL at the age of 14 to 18 years. ICSI adolescents who were informed about the mode of conception had on average significantly higher PH than the uninformed ones, but the differences were far below the limits of minimal importance. Given the increasing number of ICSI-conceived children reaching adolescence and the importance of PH and QoL, these finding are reassuring.

## Supplementary Information

Below is the link to the electronic supplementary material.

Supplementary file1 (PDF 86 kb)

## Data Availability

The datasets generated and analysed during the current study are available from the corresponding author on reasonable request.

## Abbreviations

ART: Assisted reproductive technology
ICSI: Intracytoplasmatic sperm injection
IVF: In vitro fertilisation
PH: Psychosocial health
QoL: Quality of life
