# Supplementary material for: Psychosocial health and quality of life in ICSI and naturally conceived adolescents: a cross-sectional comparison
Source: Qual Life Res. 2023 Mar 16;32(8):2223–34. doi: 10.1007/s11136-023-03382-5 (PMC10328861; doi:10.1007/s11136-023-03382-5)
Supplement: Supplementary file 1 — Supplementary file1 (PDF 86 kb) [file 11136_2023_3382_MOESM1_ESM.pdf]

Supplemental Table. Comparison of psychosocial health and quality of life in ICSI-conceived adolescents versus naturally conceived controls using adjusted linear regression models after multiple imputation – only singletons

	**Model 1: ICSI - NC No adjustment**			**Model 2: ICSI - NC Adjustment for socioeconomic and parental factors [a]**			**Model 3: ICSI - NC Adjustment for socioeconomic and parental factors [a] and adolescent factors [b]**		
	**Mean**	**95 %-CI**	**p**	**Mean**	**95 %-CI**	**p**	**Mean**	**95 %-CI**	**p**
**Psychosocial health: SDQ**									
Total difficulties (0-40 scale)	**-0.8**	**-1.4 to -0.1**	**0.016**	-0.8	**-1.5 to -0.1**	0.028	-0.7	-1.3 to 0.01	0.054
Emotional problems (0-10 scale)	**-0.4**	**-0.7 to -0.03**	**0.033**	-0.4	-0.7 to 0.01	0.057	-0.3	-0.6 to 0.03	0.071
Conduct problems (0-10 scale)	-0.1	-0.3 to 0.04	0.150	-0.1	-0.3 to 0.1	0.223	-0.1	-0.3 to 0.1	0.352
Peer problems (0-10 scale)	0.1	-0.2 to 0.3	0.605	0.0	-0.2 to 0.3	0.922	0.0	-0.2 to 0.3	0.764
Hyperactivity (0-10 scale)	**-0.4**	**-0.6 to -0.1**	**0.009**	**-0.3**	**-0.6 to -0.04**	0.028	**-0.3**	**-0.6 to -0.01**	**0.042**
Prosocial behaviour (0-10 scale)	0.0	-0.2 to 0.3	0.763	0.1	-0.2 to 0.3	0.700	0.1	-0.2 to 0.3	0.689
Impact (0-10 scale)	**-0.2**	**-0.3 to -0.04**	**0.009**	-0.1	-0.3 to 0.01	0.061	-0.1	-0.2 to 0.02	0.103
**Quality of life: KINDL (0-100 scale)**									
Total score	**2.3**	**0.7 to 3.9**	**0.005**	**2.4**	**0.6 to 4.1**	**0.008**	**2.3**	**0.6 to 4.0**	**0.007**
Physical wellbeing	**3.3**	**0.5 to 6.1**	**0.021**	**3.4**	**0.3 to 6.5**	**0.030**	**3.3**	**0.4 to 6.2**	**0.026**
Psychological wellbeing	**2.1**	**0.2 to 4.0**	**0.030**	1.9	-0.2 to 3.9	0.072	1.6	-0.4 to 3.6	0.126
Self esteem	**2.8**	**0.3 to 5.3**	**0.028**	**3.4**	**0.6 to 6.1**	**0.015**	**3.4**	**0.8 to 6.0**	**0.010**
Family	0.2	-1.8 to 2.2	0.851	0.0	-2.2 to 2.2	0.994	0.0	-2.2 to 2.1	0.246
Friends	1.1	-1.1 to 3.3	0.343	1.4	-1.0 to 3.8	0.242	1.4	-1.0 to 3.8	0.967
School	**4.4**	**1.7 to 7.0**	**0.001**	**4.4**	**1.5 to 7.4**	**0.003**	**4.6**	**1.6 to 7.5**	**0.002**

ICSI – NC: Difference between adolescents conceived with intrazytoplasmatic sperm injection and adolescents conceived naturally

[a] parental and socioeconomic factors were age of the mother at birth, the highest school degree of mother, if the mother is living single or with a partner and the family's monthly net income

[b] adolescent factors were age at time of study, sex, type of secondary school, physical exercise, smoking, alcohol consumption, body mass index category, presence of severe physical disease and presence of severe psychological disease

Bold indicates that the 95 % confidence interval does not contain 0.
